# Uveal Melanoma: Biology, Prognostication, and Emerging Therapies to Outsmart an Immune-Cold Melanoma

**DOI:** 10.3390/cancers18030432

**Published:** 2026-01-29

**Authors:** Danielle Brazel, Elizabeth Buchbinder

**Affiliations:** 1 Department of Hematology/Oncology, Scripps Health, La Jolla, CA 92037, USA; 2 Department of Medical Oncology, Mass General Brigham Cancer Institute, Harvard Medical School, Boston, MA 02114, USA

**Keywords:** uveal melanoma, BAP1, monosomy 3, tebentafusp, adoptive cell therapy, liver-directed therapy, tumor microenvironment, immunotherapy, targeted therapy

## Abstract

Uveal melanoma (UM) is a rare but highly aggressive malignancy arising from melanocytes of the uveal tract. Despite high local control rates for primary disease, half of patients ultimately develop metastatic disease with historically dismal outcomes. Unlike cutaneous melanoma, UM is characterized by a low tumor mutational burden, distinct driver mutations, and an immunosuppressive tumor microenvironment which together limit the efficacy of immune checkpoint inhibitors. Over the past decade, major advancements in molecular classification, prognostication, and therapeutic development have reshaped the clinical landscape for some patients with UM. This review synthesizes the current understanding of UM epidemiology, characteristics, prognostic biomarkers, immune biology, and contemporary management for both localized and metastatic disease. While survival gains remain modest, the rapid expansion of biologically informed and immune-based strategies offers cautious optimism for improving outcomes in this historically treatment-refractory disease.

## Simple Summary

This paper summarizes available treatment for uveal melanoma, a rare, aggressive cancer that originates in the eye. While treatments like surgery or radiation can control the tumor within the eye, about half of patients experience the spread of cancer, usually to the liver. Researchers now understand that uveal melanoma has unique genetic changes that obscure it from the immune system, which is why standard immunotherapies have limited efficacy. Recently, more tailored treatments are emerging including the bispecific antibody tebentafusp, advanced cell-based therapies, liver-focused treatments, and targeted drugs based on specific tumor biology. These advances are slowly improving the survival for patients with uveal melanoma.

## 1. Introduction

Uveal melanoma (UM) accounts for approximately 3-to-5% of melanomas and represents the most common primary intraocular malignancy in adults [1]. The disease predominantly affects older individuals with a median age at diagnosis of 62 years and occurs most frequently in individuals of Caucasian ancestry [2]. Risk factors associated with UM development include light eye color, fair skin, familial syndromes such as BAP1 and familial atypical mole and melanoma syndrome, and occupational exposure to welding.

UM arises from melanocytes within the uveal tract, involving the choroid in nearly 90% of cases, with the remainder arising from the ciliary body (5–8%) or iris (2–5%) [3,4]. Clinical presentation varies by anatomic site. Choroidal melanomas most commonly present with painless visual disturbances, including metamorphopsia and visual field defects, and larger tumors can be associated with retinal detachment. Ciliary body tumors may present later and are associated with asymmetric astigmatism or lens displacement, while iris melanomas are frequently detected earlier due to visible growth or changes in iris pigmentation.

Histopathologically, UM is classified into spindle, epithelioid, or mixed cell types. Spindle cell tumors, more common in younger patients, are associated with a more favorable prognosis. Epithelioid morphology confers a significantly higher risk of metastasis while mixed tumors demonstrate intermediate behavior [5,6].

Although local therapies achieve excellent control of the primary tumor with greater than 85% five-year survival in localized disease, metastatic relapse develops in 25–31% of patients within 5 years of diagnosis, increasing to nearly 50% of patients over longer-term follow-up [7]. The liver is involved in approximately 90% of metastatic cases, often as the first and dominant site of relapse. Other sites of metastatic spread include lungs (24%), bones (16%), and soft tissues (11%) [8]. Once metastatic disease develops, median overall survival (OS) is approximately one year, underscoring the urgent need for more effective systemic treatments [9].

UM is biologically distinct from cutaneous melanoma. It harbors a low mutational burden, usually lacks UV-driven mutational signatures, and is driven by a small set of recurrent oncogenic alterations. These features, combined with the immune-privileged ocular environment and a macrophage-dominated immunosuppressive tumor microenvironment, contribute to the limited efficacy of standard immunotherapy. Recent advances in molecular profiling, immunotherapy engineering, and liver-directed strategies are beginning to improve patient outcomes.

## 2. Molecular Pathogenesis

### 2.1. Initiating Driver Mutations

More than 80–90% of UMs harbor activating mutations in GNAQ or GNA11, which are mutually exclusive from one another [10,11]. These mutations lead to constitutive activation of multiple downstream pathways including MAPK, PI3K/AKT, protein kinase C (PKC), and YAP1 signaling. Less frequent initiating mutations include alterations in CYSLTR2 and PLCB4. These early driver mutations are not independently prognostic.

### 2.2. Secondary and Prognostic Mutations

Disease progression and metastatic risk are largely determined by later-occurring mutations. Five genes—BAP1, SF3B1, EIF1AX, GNAQ, and GNA11—define the molecular classification of melanoma. BAP1-inactivating mutations, present in approximately 47% of primary tumors and 84% of metastatic cases, are strongly associated with early metastatic spread and poor survival [12,13]. SF3B1 mutations confer an intermediate risk of metastatic disease, often with late relapse within 15 years of diagnosis. [14] EIF1AX mutations are associated with a favorable prognosis [15].

Uveal melanoma is associated with a low tumor mutational burden (TMB). The median TMB in patients with UM was 1.3 mutations per megabase, ranking in the bottom 5% out of 168 cancer types analyzed [16].

## 3. Prognostication and Risk Stratification

### 3.1. Cytogenetic Alterations

Cytogenetic profiling further refines risk stratification. Monosomy 3 and gain of chromosome 8q are hallmark features of early high-risk disease and frequently occur with BAP1 loss [13,17]. In contrast, disomy 3 and gain of chromosome 6p are associated with improved outcomes and are commonly observed in tumors with EIF1AX or SF3B1 mutations [17].

Gene expression profiling has become central to UM prognostication. As an alternative to using cytogenetic profiling for prognostication, there is a commercial test, the DecisionDx-UM assay, which is frequently used. The DecisionDx-UM assay stratifies tumors into Class 1 (low metastatic risk) and Class 2 (high metastatic risk), correlating closely with BAP1 status and long-term outcomes [18].

These molecular tools guide surveillance strategies, with high-risk patients undergoing more frequent hepatic imaging every 3–6 months for at least a decade following diagnosis.

### 3.2. CTC and ctDNA

Serum biomarkers are emerging as a complementary prognostic tool. Circulating tumor cells (CTC) are frequently detectable even in early-stage disease, reflecting the absence of a true basement membrane in the choroid. Circulating tumor DNA (ctDNA) presence has demonstrated strong prognostic and predictive value in metastatic UM and may serve as an early indicator of treatment response, particularly in patients treated with tebentafusp [19].

## 4. Tumor Microenvironment and Immune Biology

Although UM tumors contain infiltrating cytotoxic CD8+ T cells, these frequently exhibit an exhausted phenotype marked by expression of PD-1, LAG-3, and other inhibitory receptors [20,21,22,23]. The tumor microenvironment is dominated by M2-polarized macrophages, particularly in monosomy 3 and BAP1-mutant tumors, which secrete immunosuppressive cytokines and inhibit effective antitumor immunity [20,23,24].

BAP1 loss further promotes immune evasion through upregulation of PROS1 and secretion of immunosuppressive extracellular vesicles containing microRNAs. Together, these mechanisms contribute to primary resistance of immune checkpoint blockade and may also contribute to resistance to newer T-cell redirecting therapies [25].

## 5. Management of Localized Disease

Localized UM is treated with radiotherapy or enucleation, depending on tumor size and location. Episcleral plaque brachytherapy using iodine-125 or ruthenium-106 achieves excellent local control for small to medium-sized tumors and is the most commonly used eye-preserving therapy for UM. A custom radioactive plaque is sutured onto the sclera to administer trans-scleral radiation to the tumor at a dose of 62–70 Gy and is then removed after 2–7 days. The plaque should extend beyond the tumor margins by 2 mm [26]. Proton beam radiotherapy is increasingly used for larger or anatomically complex lesions but may be complicated by panuveitis and endophthalmitis in approximately 28% of patients [27]. Globe-sparing techniques have come into favor as studies have demonstrated no significant difference in survival between eye-preserving therapies and enucleation. These techniques achieve local disease control and eye salvage in 95–98% of cases [28]. Enucleation is generally restricted to advanced uveal melanomas with diameter >20 mm, thickness >12 mm, tumors causing pain or vision loss, orbital involvement, or optic nerve invasion. Although local therapies are highly effective, they do not decrease the risk of distant metastasis. Systemic therapies are currently under investigation in the neoadjuvant and adjuvant settings (Table 1).

A phase III clinical trial is evaluating the efficacy and safety of belzupacap sarotalocan (AU-011), a papillomavirus-like particle, for primary indeterminate lesions or small choroidal melanomas (NCT06007690). Darovasertinib (IDE196), a protein kinase C (PKC) inhibitor, is being studied in the neoadjuvant and adjuvant settings for GNAQ and GNA11 mutated UMs (NCT05907954) [29].

The ATOM trial is currently enrolling patients to evaluate if adjuvant tebentafusp can prevent the development of distant metastasis (NCT06246149). Recent data from a phase II multi-center study of adjuvant nivolumab and ipilimumab in high-risk uveal melanoma demonstrated a reduction in DMFS 69.1% vs. 45.1% (*p* = 0.012) when compared to matched control [30]. There are plans for further study of this regimen in the adjuvant setting.

## 6. Systemic Therapy for Metastatic Uveal Melanoma

### 6.1. Immune Checkpoint Inhibition

Single-agent PD-1 or CTLA-4 inhibitors demonstrate limited activity in metastatic UM, with objective response rates (ORRs) below 10% [31,32,33,34,35]. The PFS ranged from 2.8 to 3.8 months, and median OS ranged from 6.0 to 12.8 months.

Dual checkpoint blockade with nivolumab plus ipilimumab modestly improves response rates at the cost of higher immune-related toxicity and without consistent OS benefit [36]. Among 49 uveal melanoma patients treated with nivolumab plus ipilimumab, ORR was 16.4% with OS of 18 months. Those patients with extrahepatic metastases had better clinical outcomes compared to patients with hepatic lesions. Dual checkpoint blockade with nivolumab plus relatlimab (anti-LAG-3) resulted in an ORR of 15% in a phase II trial [37].

### 6.2. Tebentafusp and T-Cell Engagers

Tebentafusp, a bispecific T-cell engager targeting gp100 presented by HLA-A*02:01, represents a landmark advance in UM therapy. Tebentafusp binds to gp100 on the surface of melanoma cells and CD3 on the surface of T cells, bridging between them where the T cell recognizes the tumor and induces apoptosis [38]. In a randomized phase III trial, tebentafusp significantly improved OS compared with investigator’s choice therapy (pembrolizumab, ipilimumab, or dacarbazine), despite modest effects on progression-free survival (PFS). At 3-year follow-up, median OS was 21.6 months vs. 16.9 months (HR 0.68, 95% CI 0.54–0.87) [19]. A meta-analysis of 18 studies treating metastatic UM patients with tebentafusp reported an ORR of 7% (95% CI 0.06–0.09), a median PFS of 2.74 months (95% CI 2.58–2.90), and a median OS of 19.78 months (95% CI 17.79–21.77) [39].

Treatment is limited to HLA-A*02:01 positive patients. The most common side effects are cytokine release syndrome (89%), rash (83%), pyrexia (76%), pruritus (70%), and hypotension (38%). Only 2% of patients in the tebentafusp group discontinued therapy due to adverse events.

Negative ctDNA was associated with OS benefit even in cases where imaging suggested stable or progressive disease [40]. Early reduction in ctDNA at 9 weeks was strongly correlated with OS [19]. In an analysis of 183 patients on tebentafusp, 60% (*n* = 109) continued past radiographic progression for a median duration of 8 weeks [41]. Those who continued past progression had a non-statistically significant benefit in OS with a HR of 0.67 (95% CI 0.38–1.19).

### 6.3. Adoptive Cell Therapy

Adoptive cell therapies, including tumor-infiltrating lymphocytes and engineered T-cell receptor therapies targeting antigens such as PRAME, have demonstrated encouraging early activity in metastatic UM. Phase I and II studies report durable responses in a subset of patients, supporting continued investigation.

Transfer of autologous tumor-infiltrating lymphocytes have been studied in UM in a phase II study [42]. The authors found an ORR of 22.6% (*p* = 0.03) with a median DOR of 10.8 months.

The results of a phase I trial using Anzutresgene autoleucel (IMA203), a PRAME-directed T-cell therapy in previously treated advanced or metastatic cancer, were recently presented at ESMO 2025 [43]. PRAME is positive in approximately 90% of UM cases. Patients received lymphodepletion with fludarabine 30 mg/m^2^ and cyclophosphamide 500 mg/m^2^ from day –6 to day –3. Following Anzutresgene autoleucel infusion, patients received a low dose of IL-2 the following days 1–10. Sixteen UM patients were treated with a median infused dose of 3.94 × 10^9^. The ORR was 69% (*n* = 11) with a disease control rate (DCR) of 88% (*n* = 14).

CAR-T cells targeting UM-associated antigens such as PRAME or B7-H3 have demonstrated activity in pre-clinical models [44]. Co-administration with IL-2 or IL-15 further enhances CAR-T penetration into the solid tumor.

### 6.4. Targeted and Epigenetic Therapies

Targeted inhibition of PKC, MEK, MET, and other downstream effectors of GNAQ/GNA11 signaling have shown modest anti-tumor activity, though clinical benefit is often limited by resistance.

MEK inhibitors such as selumetinib and trametinib have been investigated in metastatic UM without significant clinical benefit [45,46]. Although MEK inhibitors may exhibit some antitumor activity, acquired resistance is thought to limit the PFS and OS benefit. A study targeting ERK, downstream of MEK, showed a similar lack of clinical benefit [47].

The OptimUM-01 study represents a significant advance in the treatment of HLA-A*02:01-negative metastatic UM. The phase 1/2 expansion evaluated the combination of Darovastertib, a first-in-class PKC inhibitor, with Crizotinib, a MET inhibitor, achieving a 34.1% response rate in the front line with a median OS of 21.1 months. This combination is being studied in the front-line setting for HLA-A*02:01 negative metastatic UM patients (NCT05987332). This regimen resulted in a median PFS of 7.1 months, comparing favorably to historical outcomes. The toxicity profile was manageable with most common treatment-related adverse events including diarrhea (90.9%), nausea (79.5%), peripheral edema (61.4%), and vomiting (47.7%). Grade ≥ 3 treatment-related adverse events occurred in 27.3% of participants, though treatment was discontinued in only 4.5% of participants [48]. This combination addresses a critical unmet need as approximately 50–55% of metastatic UM patients are HLA-A*02:01 negative and therefore are ineligible for the only FDA-approved systemic therapy with survival benefit, tebentafusp.

Another PKC inhibitor, sotrastaurin (AEB071), also showed modest clinical activity and tolerability in a phase I clinical trial of 153 patients [49].

Epigenetic therapies, including HDAC, EZH2, and BET inhibitors, offer a compelling strategy to reverse aggressive transcriptional programs associated with BAP1 loss and may sensitize tumors to immunotherapy. The BET inhibitor mivebresib (ABBV-075) improved survival rates by 50% in metastatic UM xenograft mouse models [50]. The PEMDAC trial combined pembrolizumab with the HDAC inhibitor entinostat [32]. This regimen led to a median PFS of 2.1 months and a median OS of 13.4 months.

### 6.5. Oncolytic Virotherapy

Oncolytic viruses are engineered to promote immunogenic cell death and local cytokine release and are an emerging therapeutic class in UM. Most commonly, these involve genetically modified herpes simplex virus (HSV) or adenovirus. The goal is to replicate many viral progenies which lyse tumor cells and stimulate systemic immune responses. Early phase trials demonstrate safety and immune activation, particularly when administered with checkpoint inhibitors or adoptive cell therapies. RP2 is an enhanced oncolytic HSV-1-expressing human granulocyte-macrophage colony-stimulating factor, a Gibbon Ape Leukemia Virus glycoprotein, and an anti-CTLA-4 antibody-like factor. In a phase I study of 17 advanced UM patients, RP2 showed an ORR of 29.4% [51]. This agent has been studied both with and without nivolumab.

The common side effects of oncolytic viruses are self-limiting and include fatigue, chills, flu-like symptoms, nausea, and fever [52]. Results from a viral surveillance program showed that 8.4% of participants reported symptoms related to HSV infection following T-VEC administration amongst close contacts [53].

### 6.6. Combination Liver-Directed Therapy with Immunotherapy

Given the hepatic tropism of UM metastases, liver-directed therapies play a central role in management of the disease. Liver-directed therapy may consist of surgical resection, SBRT, thermal ablation, transcatheter intra-arterial embolization, or percutaneous hepatic perfusion (PHP). Less invasive methods such as transarterial radioembolization (SIRT) and chemoembolization (TACE) result in a median OS of 23.3 months and 15.2 months for SIRT and TACE, respectively [54]. In a systematic review, percutaneous liver-directed therapy yielded an ORR of 39% with a median OS of 16 months [55]. PHP with melphalan is FDA-approved and achieves meaningful response rates around 60.5% and a median OS ranging from 14.9 to 21.7 months [56]. Combining liver-directed therapy with systemic immunotherapy has yielded promising results in some early-phase trials, suggesting potential synergy.

Toll-like receptor 9 (TLR9) agonist seems to improve liver lesion response to ICI therapy. The PERIO-01 phase 1 trial studied pressure-enabled drug delivery of SD-101, a TLR9 agonist, in combination with nivolumab with or without ipilimumab. At interim analysis, only one out of 47 patients included experienced a grade 3 or higher treatment-related adverse event of elevated transaminases [57]. Correlative studies showed increased serum IL-18, IFN gamma, and expansion of natural killer cells. Seven of ten patients with available samples demonstrated a decrease in ctDNA, including three with complete ctDNA clearance. The study did not proceed to phase 2, though this was not related to safety or data concerns.

**Figure 1:**
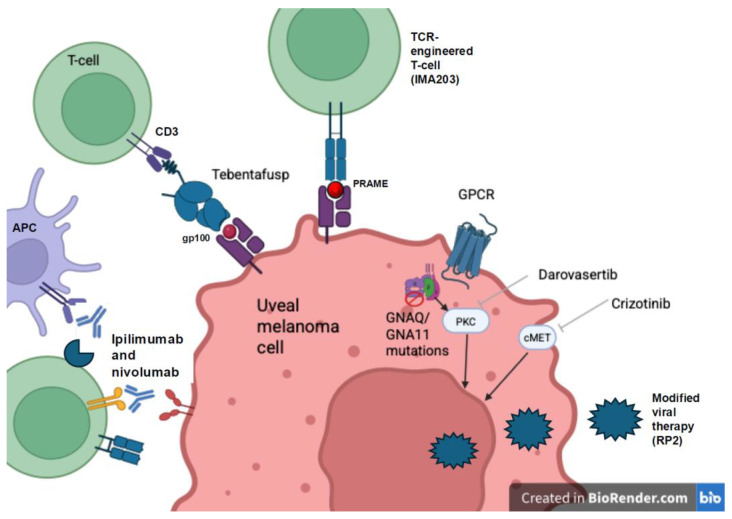
Mechanisms of action of therapies approved for and in development to treat uveal melanoma including immune checkpoint inhibition (ipilimumab and nivolumab), bispecific T-cell engager (tebentafusp), TCR engineered T cells, PKC and MET targeted therapy (darovasertib and crizotinib), and modified viral therapy (RP2). Created in Biorender. Danielle Brazel. (2025) https://app.biorender.com/illustrations/canvas-beta/6970f024f3dabba5860290f5 accessed on 23 January 2026.

Kapiteijn et al. recently presented results from the phase II CHOPIN trial at ESMO 2025. PHP was administered on weeks 1 and 7 while the combination arm also received ipilimumab 1 mg/kg and nivolumab 3 mg/kg on weeks 0, 3, 6, and 9. Combination PHP plus dual checkpoint blockade resulted in improved median PFS of 12.8 months (95% CI 9.2–15.4) vs. 8.3 months (95% CI 6.0–9.6) with a HR of 0.34 (95% CI 0.19–0.60, *p* < 0.001) [58]. Median OS improved with combination therapy at 23.1 months (95% CI 20.2–38.5) vs. 19.6 months (95% CI 15.2–21.8) with a HR 0.39 (95% CI 0.20–0.77, *p* = 0.006). Notably, the combination group experienced more grade ≥ 3 adverse events (81.6% vs. 40.5%), and patients were more likely to discontinue treatment due to adverse events (34.2 vs. 2.7%). Currently available therapies are listed in Table 2 and seen in Figure 1. Additional studies combining PHP and immunotherapy are ongoing (Table 1).

## 7. Future Directions

The therapeutic landscape of UM is rapidly evolving. Ongoing trials are evaluating adjuvant tebentafusp, novel T-cell engagers, combination targeted therapies, metabolic inhibitors, and immunotherapy combinations guided by tumor biology. Several strategies are likely to define the next phase of therapeutic development. First, combination strategies that address both immune evasion and the profoundly immunosuppressive tumor microenvironment are urgently needed, especially for patients negative for HLA-A*02:01. In addition, with novel therapies approaching approval there will need to be thoughtful sequencing studies to optimize benefit from different treatment modalities.

Approaches pairing T-cell-redirecting therapies with macrophage-modulating agents, checkpoint inhibitors beyond PD-1, or cytokine and costimulatory pathway agonists may enhance depth and durability of response. Targeting the transcriptional and epigenetic consequences of BAP1 loss represents a compelling treatment strategy to treat the most high-risk patients with systemic therapy and associated side effects. Epigenetic therapies such as BET and HDAC inhibitors may not only suppress these aggressive phenotypes but may also reprogram tumors toward increased immunogenicity, providing a rational for combination regimens.

Nanodrug delivery systems are also being explored in UM to enhance drug delivery and reduce systemic toxicity. Albumin-based nanoparticles delivering mTOR inhibitor AZD8055 achieved significant tumor reduction at lower doses in mouse models [59]. Injectable nanocomposite hydrogels have also shown sustained drug delivery, reduced tumor growth, lower recurrence, and decreased metastasis risk with a single injection [60]. Further research is needed on these drug delivery models to advance to clinical trials using human subjects.

Biomarker-driven patient selection and response monitoring represents an exciting strategy to tailor therapy based on aggressive phenotypes and treatment response. CTCs and ctDNA represent promising tools to help guide treatment selection, detect minimal disease, determine optimal duration of therapy, and enable adaptive clinical trial designs.

## 8. Conclusions

UM remains one of the most biologically distinct and therapeutically challenging tumors to treat. Despite excellent control for localized disease, those with metastatic disease have poor response to therapy and survival. Over the past decade, advances in molecular characterization including cytogenetic profiling, gene expression-based risk stratification, and identification of key prognostic mutations such as BAP1, SF3B1, and EIF1AX have reshaped understanding of disease biology and metastatic risk.

Therapeutically, UM is resistant to standard melanoma treatments due to the immune-cold microenvironment and low tumor mutational burden. The emergency of T-cell redirecting therapies, most notably tebentafusp, represents a landmark shift, demonstrating that OS benefit is achievable even in the absence of high ORR or PFS. A promising combination targeted therapy approach against PKC and MEK will hopefully break through the barrier of limited activity seen previously with similar approaches. Parallel advances in adoptive cell therapy, liver-directed approaches, and epigenetic modulation further underscore the importance of biologically informed strategies tailored to the unique immune and metabolic context of this malignancy. Future therapies should convert biologic insights into durable disease control for a broader range of patients.

## Figures and Tables

**Table 1:** Actively recruiting trials for uveal melanoma.

Trial	Phase	Intervention	Line of Therapy	Population	Targeted Enrollment	Primary Outcome(s)
NCT05607095	1	Lifileucel (LN-144 or LN-145	Any	Advanced	20	Adverse events
NCT05496686	1	Alpha particle therapy	Second line	Metastatic	16	MTD, DLT
NCT07203391	1	Tebentafusp + roginolisib	Currently on first line with tebentafusp	HLA-A*02:01 positive metastatic	8	Safety, MTD
NCT04119024	1	IL13Ralpha2 CAR-T cells	Any	Advanced or metastatic	18	Adverse events, DLT
NCT06805825	1	NN3201 c-Kit antibody drug conjugate	Received or not eligible for all standards of care	Advanced or metastatic that express c-Kit	67	DLT, adverse events
NCT04336241	1	RP2 +/− nivolumab	Received or not eligible for all standards of care	Advanced or metastatic	36	Adverse events, DLTs, MTD, RP2D
NCT06022029	1	ONM-501 +/− cemiplimab	Received or not eligible for all standards of care	Advanced	168	Adverse events, DLT
NCT04130516	1/2	LNS8801 +/− pembrolizumab	Two or fewer	Metastatic	200	MTD, RP2D, ORR
NCT03686124	1/2	IMA203/IMA203CD8	Received or not eligible for all standard of care	Recurrent or refractory that express PRAME and HLA-A*02:01 positive	375	MTD, adverse events, ORR
NCT03947385	1/2	IDE196	Any	Metastatic with GNAQ or GNA11 mutations	341	DLT, MTD, RP2D, plasma concentration, ORR, DOR
NCT06940739	1/2	IOV-3001	Second line	Advanced or metastatic	42	Adverse events, RP2D
NCT06961357	1/2	CD40L-augmented TIL	Second line	Advanced or metastatic	36	Adverse events, ORR
NCT04729543	1/2	MAGE-C2 TCR T Cell	Second line	Metastatic	20	MTD, objective anti-tumor response
NCT06626516	1/2	Tebentafusp + liver directed therapy	First or second line	HLA*A02:01 positive metastatic	109	Adverse events, PFS
NCT07136181	1/2	NBM-BMX	Any	Metastatic	36	DLT, MTD, ORR
NCT07063875	1/2	Tebentafusp + IL-2	After progression on tebentafusp	HLA-A*02:01 positive metastatic	8	safety
NCT06932757	2	Quisinostat	Adjuvant	High risk localized	63	DMFS
NCT06070012	2	Tebentafusp	First line	HLA-A*02:01 positive metastatic	44	ctDNA response
NCT05077280	2	Radiotherapy + Opdualag	Prior PD1 or tebendafesp allowed	Metastatic	40	Adverse events
NCT05907954	2	Darovasertib	Neoadjuvant or adjuvant	Localized	160	Adverse events, rate of enucleation, change in radiation dose, CBR
NCT03467516	2	Non-myeloablative lymphodepleting conditioning for autologous TIL	Any	Metastatic	47	ORR
NCT06717126	2	roginolisib	Following immunotherapy and tebentafusp if HLA-A*02:01 positive	Advanced or metastatic	85	OS
NCT05524935	2	Olaparib + pembrolizumab	First line	Advanced	37	ORR
NCT06121180	2	Cemiplimab + ziv-aflibercept	Any	Metastatic	32	ORR
NCT07057596	2	Tebentafusp	Neoadjuvant	Metastatic with resectable liver metastases only	19	pCR rate
NCT06627244	2	Tebentafusp + Yttrium-90 (Y-90)	Any	HLA-A*0201 positive metastatic		PFS, adverse events
NCT06414590	2	Tebentafusp	Neoadjuvant	HLA-A*02:01 positive localized	19	Regression in 20% of patients
NCT05987332	2/3	Darovasertinb + crizotinib	First line	Metastatic	420	PFS, OS
NCT06581406	2/3	RP2 + nivolumab	Checkpoint-inhibitor naive	Metastatic	280	PFS, OS
NCT06519266	3	PHP + ipilimumab 1 mg/kg + nivolumab 3 mg/kg	Treatment naive or progression on tebentafusp	Metastatic	40	PFS
NCT05502900	3	Melatonin	Adjuvant	Localized	100	Rate of metastases
NCT06246149	3	Tebentafusp	Adjuvant	High risk localized	290	RFS
NCT07015190	3	Darovasertib	Neoadjuvant	Localized	520	Vision loss, rate of enucleation
NCT06007690	3	Belzupacap sarotalocan (AU-011)	Treatment naive	Localized primary indeterminate lesions or small choroidal melanoma	100	Time to tumor progression

CBR: clinical benefit rate; CtDNA: circulating tumor DNA; DLT: dose-limiting toxicities; DMFS: distant metastasis-free survival; DOR: duration of response; MTD: maximum tolerated dose; ORR: objective response rate; OS: overall survival; pCR: pathologic complete response; PFS: progression-free survival; PHP: percutaneous hepatic perfusion; PRAME: preferentially expressed antigen in melanoma; RFS: recurrence-free survival; RP2D: recommended phase 2 dose.

**Table 2:** Treatments for metastatic uveal melanoma.

Regimen	ORR	PFS (Months)	OS (Months)	Most Common AEs
Single agent PD-1 or CTLA-4	10%	2.8–3.8	6.0–12.8	Fatigue, diarrhea, rash, pruritus
Nivolumab + ipilimumab	16.4%	5.5	19.1	Fatigue, diarrhea, rash, pruritus
Nivolumab + relatlimab	15%	Unknown	unknown	Fatigue, diarrhea, rash, pruritus
Tebentafusp	7%	2.74	19.8	CRS, rash, pyrexia, pruritus, hypotension
Anzutresgene autoleucel (IMA203)	69%	8.5 months	unknown	Cytopenias, cytokine-release syndrome
Darovasertib + crizotinib	34.1%	7.1	21.1	Diarrhea, nausea, edema, acneiform dermatitis, fatigue
Pembrolizumab + entinostat	14%	2.1	13.4	Transaminitis, neutropenia, rash, nausea, fatigue
RP2 +/− nivolumab	29.4%	unknown	unknown	Fatigue, chills, flu-like symptoms, nausea, fever
PHP + ipilimumab + nivolumab	38%	12.8	23.1	Transaminitis, anemia, cutaneous toxicities

## Data Availability

No new data were created or analyzed in this study. Data sharing is not applicable to this article.
